# Chicken IFN Kappa: A Novel Cytokine with Antiviral Activities

**DOI:** 10.1038/s41598-017-02951-2

**Published:** 2017-06-02

**Authors:** Diwakar Santhakumar, Munir Iqbal, Venugopal Nair, Muhammad Munir

**Affiliations:** The Pirbright Institute, Woking, Surrey GU24 0NF United Kingdom

## Abstract

Interferons (IFNs) are essential components of the host innate immune system and define first-line of defence against pathogens. In mammals, several type I IFNs are identified, however, only limited data is available on the repertoire of IFNs in avian species. Here we report the characterization of chicken IFN-κ (chIFN-κ) near the type I IFN locus on the sex-determining Z chromosome. Genetic, evolutionary and syntenic analyses indicate that chIFN-κ is a type I IFN with conserved genetic features and promoter binding sites. chIFN-κ regulated the IFN-stimulated response element signalling pathways and activated a panel of IFN-regulated genes, antiviral mediators and transcriptional regulators. Priming of chicken primary fibroblasts and tracheal organ cultures with chIFN-κ imparted cellular protections against viral infections both *in vitro* and *ex vivo*. To determine whether chIFN-κ defines the antiviral state in developing chicken embryos, we used replication-competent retroviral RCAS vector system to generate transgenic chicken embryos that constitutively and stably expressed chIFN-κ. We could demonstrate that chIFN-κ markedly inhibited the replication of avian RNA viruses *in ovo*. Collectively, these results shed the light on the repertoire of IFNs in avian species and provide functional data on the interaction of the chIFN-κ with RNA viruses of poultry and public health importance.

## Introduction

Interferons (IFNs) are a family of pleiotropic and functionally related cytokines that play central roles in the cellular antiviral defence, antitumor, antiproliferative and immunomodulatory activities^1–3^. Based on structural and functional characteristics, all IFNs are divided into three sub-types; those that bind IFN-α receptor 1 (IFNαR1) and IFNαR2 (type I IFNs), those that interact with receptors complexes of IFNγR1 and IFNγR2 (type II IFNs) and those that bind with heterodimeric receptor complex of IL28Rα and IL10Rβ (type III IFNs or IL28/29)^4^. Although, the IFN-γ and IFN-λ (IFN-λ1, IFN-λ2, IFN-λ3 and IFN-λ4) are the solo member of the type II and III IFNs respectively, at least nine type I IFNs are reported in different mammals^5^.

These IFNs are crucial to prime innate immunity, however, their expression and antiviral activities are cell and virus-dependent mainly due to the expression of cognate receptors and importance in a particular organ or system^4, 5^. Specifically, type I IFNs (IFN-α and IFN-β in case of chicken) are primarily produced in fibroblasts whereas the antiviral actions of type III IFNs are mainly restricted to epithelial cells of respiratory and gastrointestinal tracts^6, 7^. Despite the dedicated roles of IFNs, certain levels of functional and regulatory overlaps have been identified in type I and III IFNs. Upon ligand recognition, IFNs activate the Janus kinase/signal transducers and activators of transcription (JAK-STAT) pathway by phosphorylation of STAT1 and STAT2. This STAT1-STAT2 heterodimerization leads to the recruitment and phosphorylation of IFN regulatory factor 9 (IRF9, not yet identified in chicken) to constitute interferon-stimulated gene factor 3 (ISGF3) in mammals^3, 8, 9^. Upon nuclear translocation, the ISGF3 binds to IFN-stimulated response elements (ISREs) and transcriptionally activates hundreds of ISGs (such as Mx, PKR, 2′-5′ OAS, IFITMs, IFITs), which play fundamental roles in a wide range of cellular activities, including transcriptional and translational regulation of immune responses. The collective actions of these ISGs counteract viral replication, and provide an antagonistic environment to limit virus propagation and spread^3, 9, 10^.

Type I IFNs have a conserved intronless structure, co-localized in a specific locus in the chromosomes and are considered to have arisen from gene duplications^11, 12^. Several type I interferons (IFN-α, IFN-β, IFN-ω, IFN-ɛ, IFN-κ, IFN-δ, IFN-ζ and IFN-τ) have been identified in mammals with distinct and characteristics tissue distributions and functions^13^. However, only two type I IFNs (IFN-α and IFN-β) have been characterized in chicken and other members of aves^9^. Studies on the diverse functions of type I IFNs in innate and adaptive immunity are rapidly mounting^14, 15^ and most of these investigations focus on the IFN-α and IFN-β.

Since chickens mount a potent antiviral state similar to mammals, it is plausible that chicken carry a mammalian-like broader repertoire of type I IFN genes (*n* = *9*). Previously, several immunogenetic investigations have suggested that chicken type I IFN spectrum is represented by additional IFNs genes^16–18^. However, conclusive studies on the genetics, functional or evolution of such chicken IFNs genes are lacking. Moreover, there are emerging evidences on genetics and immunological differences between avian species, and that they mount evolutionary diverse innate immune responses^9^. Understanding these differential host responses may provide foundations to study evolutionary and functional genomic backgrounds of virus-host interactions in class *Aves* especially in chickens, which are notoriously been associated with emergence of influenza viruses^19, 20^. In an effort to determine the dynamics of immune responses in birds, here we report the identification and characterization of IFN-κ in different avian species including chicken. Chicken IFN-κ (chIFN-κ) proteins were used in functional assays to investigate the initiation of IFN signalling pathways and antiviral activities against avian RNA viruses both *in vitro* and *ex vivo*. Employing retroviral gene transfer vector system, RCAS^21^, we generated chIFN-κ expressing transgenic chicken embryos and demonstrated its antiviral potential *in ovo*. These analyses provided evidences on the presence of a novel and previously uncharacterized IFN gene in aves.

## Results

### Isolation and genomic architecture of chIFN-κ gene

**Figure 1:**
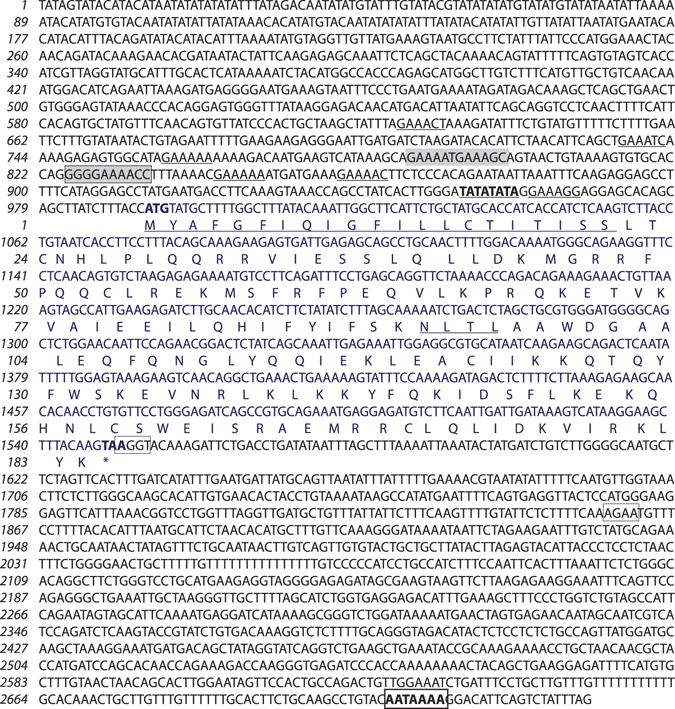
Structure of chIFN-κ CDS and promoter. A ~3.0 Kb genomic sequence including promoter and the CDS of chIFN-κ is shown in the reverse complement order. The ORF of the chIFN-κ is translated in uppercase and putative binding sites for IRF7 and NF-κB transcription factors are shaded in grey. The TATA box is bold and underlined, whereas six GAAANN sites are underlined. The splice donor and accepter sites are boxed, and putative polyadenylation signal is boxed and is in bold. A predicted signal peptide and N-glycosylation site are underlined within the amino acid sequence of the chIFN-κ.

Using the human IFN-κ gene as query, BLAST analysis revealed a stretch of sequence on the sex-determining chicken Z chromosome at the junction of AC2319722 and AC234262.3 contigs. This region maps to the chicken type I IFN locus. The location of IFN-κ gene was syntenic with the human chromosome 9p21 which encodes the type I IFN gene cluster including human IFN-κ^11, 22^. The predicted sequence for the putative chIFN-κ gene was cloned and sequenced to determine the genomic structure of chIFN-κ. Sequences from 8 different chicken lines were submitted to the GenBank and are available under the accession numbers from KR817814 to KR817821. Except for synonymous mutations, the coding DNA sequence (CDS) obtained from genomic DNA of different chicken lines was similar to the sequence available in the GenBank (https://www.ncbi.nlm.nih.gov/nuccore/HQ267515), reported previously by Andrew Bean’s group (CSIRO, Australia). Analysis of transcriptomics datasets from Marek’s disease virus (MDV)-infected CEFs, splenocytes, and immune (CD4 naïve) cells showed induced transcript expression compared to mock infected CEFs (Fig. 1SA). Consensus sequences from these datasets (transcriptomics data, sequences available in Ensembl and GenBank, and from gene characterization) revealed that the chIFN-κ gene encodes a putative protein of 184 amino acids and shows characteristic features for type I IFN including a putative signal peptide, N-glycosylation (^93-^NLTL^-96^) and an IFN-α receptor binding site (Fig. 1). However, chIFN-κ was encoded in the opposite orientation on the Z chromosome compared to the IFN-α and IFN-β genes and is approximately 27 Mb apart from these genes (Fig. 1SB).

In addition to the putative transcript region for chIFN-κ, a ~1.0 kb sequence upstream and ~1.0 kb sequence downstream was analysed. The analysis not only confirmed the ORF identified in the cDNA sequence but also revealed the presence of splice donor and acceptor sites leading to an intron in the 3′ untranslated region of the CDS. Similar to the human homologue^11^ and unlike mouse IFN-κ^23^, the second exon was observed immediately after the stop codon. Inspection of the promoter region predicted an IRF7 binding site (5′-GAAANNGAAANN-3′) which is a characteristic for virus-inducible genes^24^ (Fig. 1). Downstream to the IRF7 binding site, a modified nuclear factor kappa-light-chain-enhancer of activated B cells (NF-κB)-like motif (5′-GGGRNNHHCC-3′ where R indicates G, H indicates A, and N indicates any nucleotide), which differs at position 7 and 8 from the decameric consensus sequences of NF-κB was observed. An atypical TATA box (AT-rich sequence in contrast to TATAA) was noticed within the 40 bps of the putative transcriptional start site^25^. Interestingly, six GAAANN elements were predicted within the 400 bp of the translational start site. These elements have been reported curial for the virus-induced type I IFN-mediated binding of the IRF family members^26^.

### Genetic analysis and nomenclature of chIFN-κ

**Figure 2:**
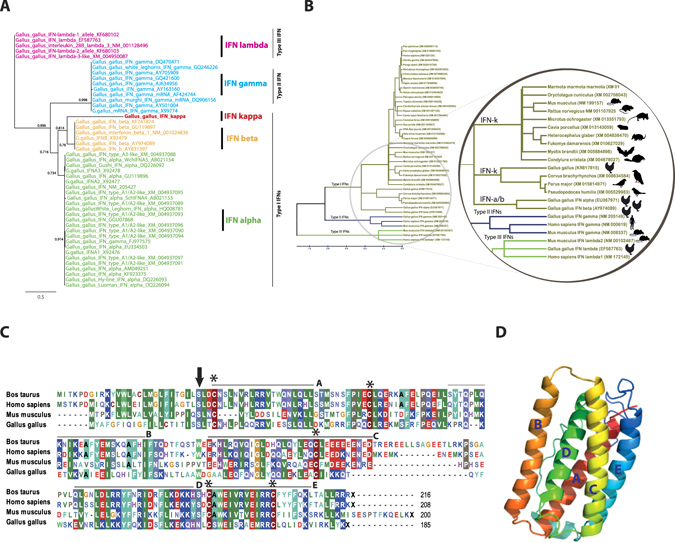
Genetic analysis of IFN genes. (**A)** Clustering patterns of all known chicken type I, II and III IFN genes by neighbour-joining method with bootstrap value at *n* = 2000. The tree was constructed with only ORF sequences including stop codon, and NCBI accession numbers are shown in each of the analysed sequences. Bootstrap values less than 50% were not shown. (**B)** Phylogenetic tree of type I, II and III IFNs from different mammals, aves and rodents. The inset highlights the association of chicken IFN-κ with closest avian and non-avian species. NCBI accession numbers used for the analysis are shown in parentheses in each of the sequence in the tree. (**C**) Alignment of the chicken IFN-κ with IFN-κ protein sequences of bovine (NM_001206423), human (NM_020124) and mouse (NM_199157). Amino acid sequences were aligned using ClustalW algorithm and colour represents similarities in the amino acid properties. Putative cleavage signal is marked with down head arrow and five conserved cysteines are labelled with asterisk. Sequences overlaid with lines represent α-helices (A to E) corresponding to human IFN-κ. (**D)** Predicted three-dimensional structure of chicken IFN-κ where five α-helices are annotated.

Phylodynamic analysis of homology-based coding sequences of all known types of chicken IFNs indicated that chIFN-κ did not group with any of the currently known chicken IFNs and it defined a new cluster with high confidence (bootstrap value of >80%) (Fig. 2A). This distant branching pattern shown close association with type I IFN compared to type II or III chicken IFNs. Data mining of recently reported genomes of more than 40 avian species^27^ showed a homologue of chIFN-κ gene encoded in at least 25 investigated birds (Fig. 2SA). In comparison to IFNs from vertebrates, the chIFN-κ clustered within previously reported IFN-κ group in mammals and constituted a sister group with other avian species (Fig. 2B). This sub-group was clustered apart from IFN-α and IFN-β within the main type I IFN group.

A pairwise BLAST analysis revealed that chIFN-κ gene is 37.0%, 27.0% and 35.0% homologues to recently characterized human^11^, mouse^23^ and bat^28^ IFN-κ, respectively. This amino acid similarity is indicative throughout the length of the chIFN-κ protein (Fig. 2C) and the similarity between IFN-κ from different mammalian and avian species was significantly higher than the similarity between chicken IFN-κ and chicken IFN-β, -α, -λ and -γ (Fig. 2SB). However, within chicken IFN genes, the maximum identity of chIFN-κ gene was observed with chIFN-β (25.8%) whereas minimum identity was noticed between chIFN-κ and chIFN-γ genes (8.6%) (Fig. 2SC). Based on the homology and modelling of the mature chIFN-κ protein with the corresponding IFNs in mammals, five highly conserved cysteines (Cys) residues were identified (Fig. 2C). Structurally, Cys3 was modelled to form a disulfide bond with Cys100 and Cys32 was proposed to be establishing disulfide bond with Cys138, whereas Cys150 would be an unpaired cysteine. Furthermore, consistent to crystal structures of other type I IFNs, five putative alpha helices were revealed in the 3D structure of chIFN-κ protein (Fig. 2D). Taken together, these comparative genetic characterizations indicate that IFN-κ gene is a previously uncharacterized class of IFNs in aves and shares characteristic features of type I IFNs.

### The chIFN-κ initiates IFN signalling and ISGs induction in CEFs

**Figure 3:**
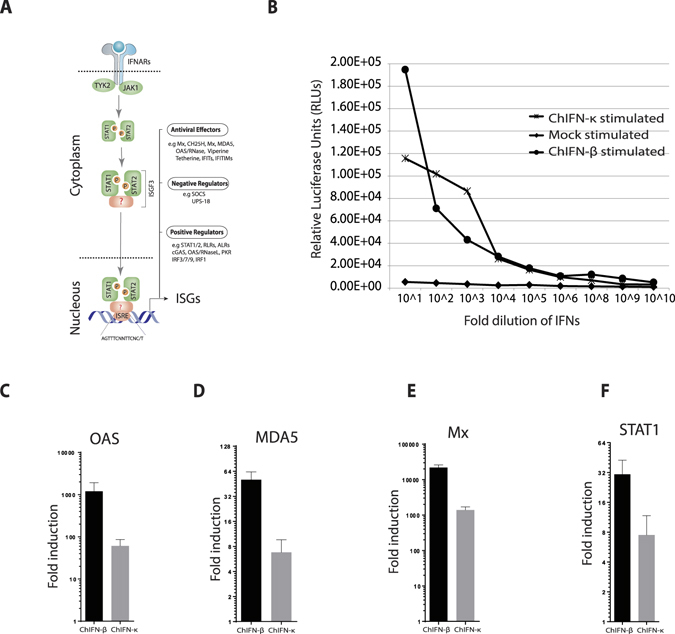
The chIFN-κ induces IFN signalling and transcriptional activation of ISGs in CEFs. (**A)** Type I IFNs interact with the IFN receptor complex and initiate the formation of ISGF3 transcription factor through JAK-STAT pathway. Induction of ISGs, which determine the antiviral state of the host, is dependent on the ISGF3 nuclear translocation and interaction with the ISRE promoter (AGTTTCNNTTCNC/T, where N denotes any nucleotide). (**B**) CEF cells were transfected with the Mx-ISRE-Luc (150 ng) and RL-SV40 (10 ng) plasmids for 24 hours in triplicate before stimulation with the serial dilution of chIFN-κ, chIFN-β (expressed in heterologous HEK-293) or media (mock-stimulated) only. The relative luciferase units were determined by the removal of the background and the ratio of Renilla and Firefly luciferase values. (**C–F)** Transcriptional profiling of the OAS **(C)**, MDA5 **(D)**, Mx **(E)** and STAT1 **(F)** genes in the chIFN-κ and chIFN-β treated CEFs. Mock treated cells were used to calculate the fold induction, and mRNA levels of these genes are expressed relative to 28 S rRNA levels. The shown fold induction is average of two independent experiments performed in triplicate.

Amongst the roles of IFNs, antiviral activities of these cytokines are the most prominent and well defined^1^. For antiviral actions, all type I IFNs signal through a common IFNAR1 and IFNAR2 receptor complex to initiate the JAK-STAT signalling pathway, which culminate in the activation of ISGF3. Translocation of ISGF3 leads to transcriptional activation of ISGs through ISRE promoter which establishes an antiviral state^9^ (Fig. 3A). To determine whether chIFN-κ mediates a likewise activation of signalling pathway, the chIFN-κ gene was expressed in heterologous mammalian (HEK 293) and in insect S2 cell culture system. The chIFN-κ showed a clear and dose-dependent activation of the Mx-ISRE promoter in DF-1 cells transiently transfected with plasmid containing tandem copies of an Mx ISRE element upstream to the luciferase reporter gene. Except the undiluted concentrations, the promoter activation by the chIFN-κ was comparable to previously characterized chIFN-β^29^, and was markedly higher than the mock-stimulated control (Fig. 3B).

To assess the potential of chIFN-κ induced activation of ISGs, similar to other IFNs, primary CEF cells were treated with the chIFN-κ and chIFN-β or were left untreated. After 12 hours of stimulation, total RNA was harvested and subjected to quantitative real-time PCR for a subset of ISGs which are previously demonstrated to be mediated by type I IFNs^30^. Both chIFN-κ and chIFN-β induced the expression of mRNA levels of OAS, MDA5, and Mx genes which are relatively well-characterized antiviral pathways in chickens^9^ (Fig. 3C–E). In addition to ISGs, mRNA level of STAT1 transcription factor, which plays crucial roles in the establishment of antiviral states, was activated (Fig. 3F). Taken together, chIFN-κ activates the ISRE promoter and induces the transcriptional activation of ISGs possibly through the activation of STAT1, which is a shared-component of type I, II and III IFN signalling pathways.

### Antiviral activities of chIFN-κ in CEFs and tracheal organ cultures

**Figure 4:**
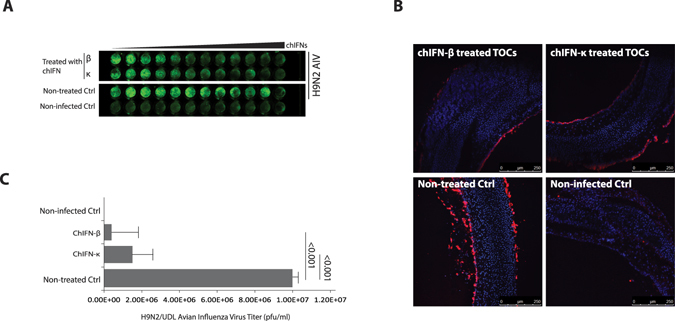
The chIFN-κ mediates an antiviral state *in vitro* and *ex vivo*. (**A**) CEF cells stimulated with increasing concentrations of chIFN-κ, chIFN-β (expressed in heterologous HEK-293) or media only (non-treated Ctrl), were infected with AIV (H9N2/UDL/08) or were left uninfected (non-infected Ctrl). Immunostaining of the NP protein of AIV correlates the virus replication in infected cells and antiviral activities of chIFN-κ and chIFN-β. (**B**) TOCs were stimulated for antiviral response with 1000 Units of chIFN-κ and chIFN-β before infection with AIV (H9N2/UDL/08). Immunostaining of the NP protein of influenza virus indicate the cellular replication levels of influenza viruses in IFNs treated TOCs compared to non-infected and non-treated controls. (**C**) Quantitative measurement of AIV ((H9N2/UDL/08) in TOCs supernatants. The data represents 2 or 3 independent experiments.

To demonstrate whether chIFN-κ-induced gene expression establishes an antiviral state, CEFs were treated with serial dilution of chIFN-κ, chIFN-β or control medium before challenge with an avian influenza virus, UDL/08/H9N2. In-cell Western blotting demonstrated that chIFN-κ, similar to the chIFN-β, established an antiviral state for the AIV in a dose-dependent manner compared to the non-stimulated control (Fig. 4A).

Next, we investigated the antiviral potential of chIFN-κ *ex vivo* using tracheal organ culture (TOC) model system. TOCs prepared from 20 days old chicken, were stimulated with IFNs or were left untreated before challenge with UDL/08/H9N2. Immuno-staining of the NP, a structural protein of AIV, in TOCs sections show suppressed virus replication in both chIFN-κ- and chIFN-β-treated organs compared to mock-stimulated chicken TOCs (Fig. 4B). To assess the quantitative virus replication and the magnitude of release of infectious virus particles, supernatants from TOCs were collected and virus quantification was performed using plaque assay in MDCK cells. In accordance to results demonstrated in Fig. 4B, a significant (p < 0.001) reduction in the virus release was observed in IFNs treated organs compared to mock-treated control (Fig. 4C). These findings demonstrate that the chIFN-κ is a potent inhibitor of virus replication *in vitro* and *ex vivo* which is one of the best studied features of IFNs.

### Stable expression demonstrates antiviral activities of chIFN-κ gene against RNA viruses

**Figure 5:**
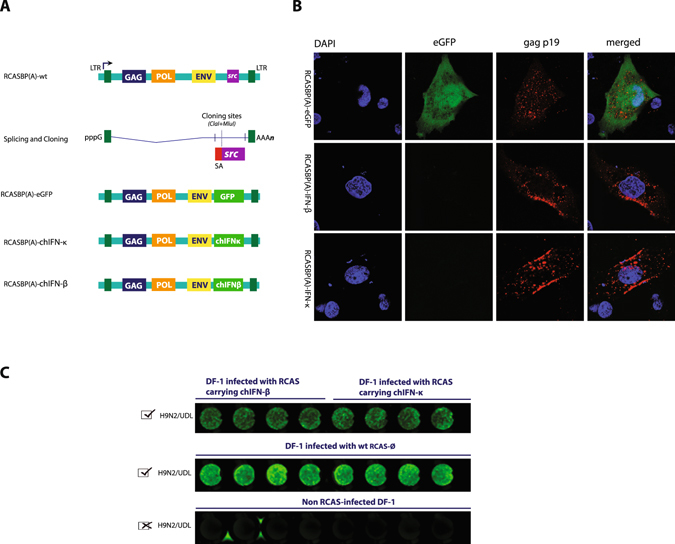
Construction and rescue of chIFN-κ, chIFN-β and GFP expressing recombinant RCASBP(A) viruses. (**A**) Schematic illustration of the proviral DNA, and viral genes (gag, pol, env and src). The expression of transgenes is initiated by the viral LTR. Host DNA-dependent RNA polymerase transcribes viral mRNA that is capped (indicated by pppG) and polyadenylated (AAAn) at their 5′ and 3′ ends, respectively. Foreign genes of length lesser than 2.8 kb can be cloned between ClaI and MluI sites that replace most of the *src* gene. The expression of the foreign gene is mediated via the *src* splice accepter (SA). Construction of RCASBP(A)-eGFP, RCASBP(A)-chIFN-κ and RCASBP(A)-chIFN-β was performed by replacing *src* gene with GFP and chIFN-β and chIFN-κ cDNA, respectively. (**B**) Stable cell lines constitutively expressing either eGFP (upper panel), chIFN-β (middle panel) or chIFN-κ (lower panel) were stained for RCASBP(A) viral structural protein, *gag*, to demonstrate the effective rescue and replication of the viruses. (**C**) DF-1 cells stably expressing RCASBP(A)-delivered chIFN-β and chIFN-κ were infected with an moi 1 of AIV (UDL/08/H9N2). Cells were then stained for the AIV-specific NP antibodies to demonstrate the chIFN-β and chIFN-κ mediated inhibition.

We next constructed replication-competent ALSV long terminal repeat (LTR) with a splice acceptor (RCAS) to constitutively express chIFN-κ (Fig. 5A). Similarly, RCAS expressing chIFN-β and GFP were generated to compare the antiviral actions of chIFN-κ, and to monitor the rescue and replication kinetics of RCAS, respectively. Transfection of RCASBP(A)-eGFP vectors into DF-1 fibroblasts demonstrated the successful rescue of viruses and spread to all DF-1 cells within four days (Fig. 3SA). Due to their efficient replication competency, approximately 10% of transfected cells generated sufficient progeny viruses that rapidly spread between cells and fully saturated the infectivity within three cell passages. To further verify the rescue of RCASBP(A)-eGFP virus and to monitor the replication competency of RCASBP(A)-chIFN-κ and RCASBP(A)-chIFN-β, RCAS-infected DF-1 cells were stained for the *gag* structural protein of the RCAS virus. As depicted in the Fig. 5B, the insertion of chIFN-κ and chIFN-β cDNA did not affect the rescue of the virus, and virus replication was comparable to the GFP expressing RCAS virus. In order to monitor the induction of innate immune genes by retroviruses, the transcription of ISGs were monitored in cells infected with wt-RCASBP(A), RCASBP(A)-chIFN-κ and RCASBP(A)-chIFN-β. As expected, RCASBP(A)-cytopathic effects were not observed and a non-significant innate immune responses were inducted observed by the wild-type RCASBP(A) viruses (Fig. 3SB) compared to RCASBP(A)-chIFN-κ and RCASBP(A)-chIFN-β. These results exclude the possibility of RCAS-induced immunity and subsequent establishment of antiviral state. Additionally, there are evidences that due to integration of transgene into the host genome, the expression of gene may occurs independent of the virus replication^31^.

We used these RCASBP(A)-infected chicken fibroblasts to assess the virus inhibitory roles of chicken IFNs against UDL/08/H9N2 virus. As depicted in the in-cell Western blotting, the expression of chIFN-κ through RCASBP(A)-chIFN-κ resulted in the establishment of an antiviral state in chicken cells compared to the control RCASBP(A)-wt infected or mock-infected cells (Fig. 5C). Comparable antiviral effects were observed with chIFN-β stimulated positive control. Additionally, both chIFN-κ and chIFN-β inhibited the release of UDL/08/H9N2 virus significantly higher whereas the wt RCAS was unable to restrict virus replication (Fig. 5C). These results demonstrate that chIFN-κ carries antiviral activities in chicken fibroblasts and these antiviral actions are comparable to previously reported antiviral effects of chIFN-β. Additionally, these results further confirm the use of RCASBP(A) vector system as an effective, safe, convenient and highly proficient gene delivery system to express the transgene *in vitro* cell culture models to study innate immune genes.

### Embryonated chicken eggs transgenically expressing chIFN-κ inhibit RNA viruses

**Figure 6:**
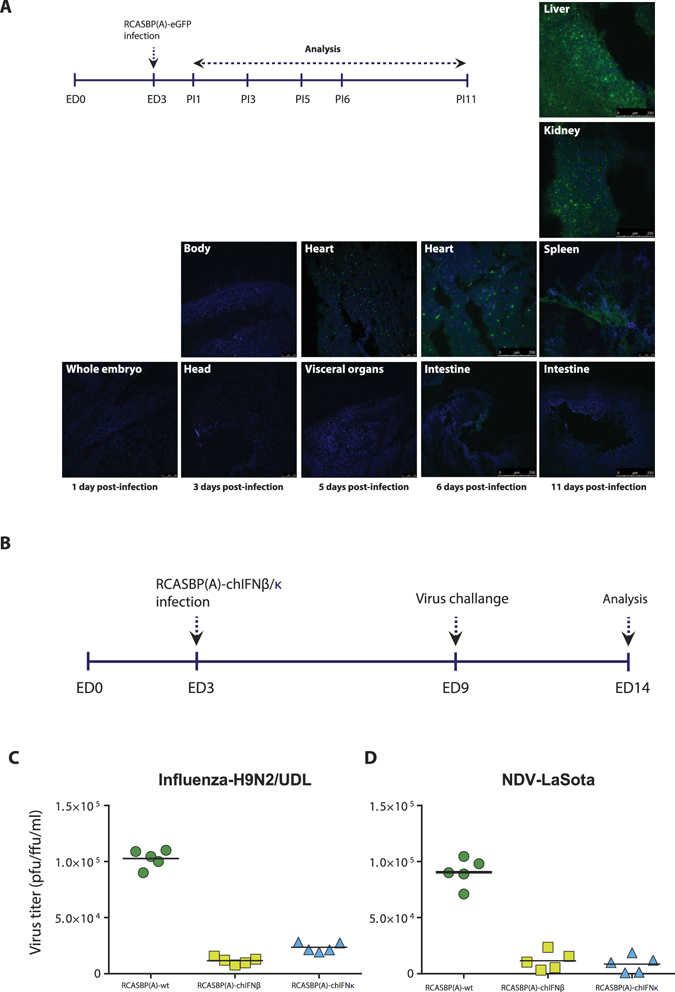
Spatiotemporal expression dynamics of the transgene in developing embryos, and antiviral activity of chIFN-β and chIFN-κ *in ovo*. (**A**) Using experimental layout, depicted in the schematic illustration in the inset, expression of the GFP reporter gene indicated the embryo-wide distribution. After infection of eggs on third day of embryonation (ED), the expression of the GFP was detected as early as 6 days post-infection (PI) and most organs of the embryos were positive for the transgene at day 11 post-infection. Representative images from each time points are shown, and all images are shown at x40 magnification. (**B**) Experimental layout for the generation of transgenic embryos and analysis of the antiviral effects of IFNs *in ovo*. (**C)** Replication of both influenza and ND viruses was markedly compromised in the allantoic fluids collected from eggs expressing RCASBP(A)-mediated chIFN-β and chIFN-κ compared to RCASBP(A)-wt virus. Each dot, circle or triangle represents individual egg and the cross-lines show the mean in each group.

In order to assess the biological activities of chIFN-κ *in ovo*, we generated mosaic-transgenic chicken embryos constitutively expressing chIFN-κ and chIFN-β, and monitored the replication of two economically important poultry viruses, avian influenza and NDV. To demonstrate the potential of RCASBP(A) in delivering genes *in ovo* and to monitor the expression dynamics of exogenous genes, we first generated reporter transgenic embryos using RCASBP(A)-eGFP and studied the expression and organ distribution of eGFP for 2/3rd life of the chicken embryo (Fig. 6A). A time-course monitoring of the marker genes indicated that transgenes expressed as early as 6 days post-infection and lasted until the end of the experiment on 14^th^ days of embryonation. Since different organs were not easily markable at this early stage of embryonation, we sectioned the entire embryo on day 3, 5 and 6 post-infection. At day 11, when most of visceral organs can be identified and mounted separately, marker protein was visualized in all tested organs including liver, kidney, spleen and intestine (Fig. 6A). In organs such as brain and beak, a relatively lower expression of the fluorescent protein was observed. However, the expression of reporter transgene was markedly higher in tissues that were abundant with the endothelial cells such as liver and kidney (Fig. 6A). In agreement to previous findings^32, 33^, these results demonstrate that RCAS vector system can be used effectively to express transgene *in ovo* and this gene delivery system can be exploited to monitor biological activities of constitutively expressed genes.

We next applied this vector system to generate mosaic transgenic chicken embryos that express chIFN-κ and chIFN-β by inoculating chicken embryos with RCASBP(A)–chIFN-β and RCASBP(A)–chIFN-κ or with RCASBP(A)-wt infected DF1 cells in fertilized eggs on the embryonic day 3 (Fig. 6B). To monitor the influence of constitutively-expressed chIFN-β and chIFN-κ on the replication kinetics of viruses, embryos were inoculated with UDL/08/H9N2 and NDV-LaSota in the allantoic cavity on day 9 of embryonation (Fig. 6B). Quantitative analysis of virus replication at day 14 of embryonation and 3 days post-virus challenge indicated a decrease in both UDL/08/H9N2 (Fig. 6C) and NDV-LaSota (Fig. 6D) titres compared to embryos that were infected with RCASBP(A)-wt. Collectively, these results confirm the antiviral activity of chIFN-κ *in ovo*.

## Discussion

Owing to differences in several innate immune checkpoints, all members of the order Galliformes including chickens respond to viruses differently compared to members of the order Anseriformes such as ducks^9^. Mechanisms that underline these differences and how these differences impact the innate immune inductions are not yet fully elucidated. Recent genetic and functional studies have demonstrated unique features of chicken (and possibly other Galliformes) innate immune system such as absence of RIG-I, IRF3 and IRF9^9^. However, data on functional antiviral molecules that are released in chickens following viral infection is still largely unclear. Here, we report the genetic and functional characterization of IFN-κ in chicken and have identified gene homologs in different avian species. Based on chromosomal location, genetic clustering patterns and structural features, IFN-κ is closely related to type I IFNs. However, in contrast to the rest of type I IFNs, IFN-κ gene has been identified outside the type I IFN locus both in mammals^11, 23, 28^ and in chicken (this report). Phylogenetic analysis of chIFN-κ gene revealed that it is grouped separately from other mammals and constituted an outlier within type I IFN cluster in chicken and other avian species. Further evolutionary analysis of chIFN-κ with corresponding genes from other species failed to identify ortholog of chIFN-κ, proposing that it may have evolved separately from rest of the type I IFNs^11, 12^. Additionally, the sequence conservation and diversification of IFN-κ genes in different avian species and in mammals, respectively, implies the presence of evolving and adaptable IFNs in aves.

Type I IFNs mediate numerous cellular activities through the activation of a wide spectrum of ISGs including inhibition of tumour proliferation, enhancement of antigen expression and regulation of natural killer cells^1–3, 9, 10, 34^. Amongst all roles, antiviral activity of type I IFNs is the best characterised function. Therefore, we primarily focused on the antiviral potential of this newly identified class of type I IFNs in avian species. We chose to monitor the antiviral impacts of chIFN-κ against NDV and AIV, since both viruses are economically important pathogens in poultry industry and AIVs are associated with infections of public health significance^20^. Owing to replication potential of both viruses in embryonated chicken eggs, correlation of *in vitro* antiviral activities of chIFN-κ was possible with the corresponding effects *in ovo*. Using expressed chIFN-κ, we demonstrated that the transcriptional activation of ISG, as was evidenced by the ability of chIFN-κ to activate Mx ISRE, determine the antiviral potential similar to previously characterized type I IFNs^30^. Albeit weaker than chIFN-β, initial transcriptional profiling of ISGs in CEFs implied that chIFN-κ elicit antiviral activities by regulation of at least OAS, MDA5 and Mx-mediated antiviral pathways. Additionally, chIFN-κ up-regulated the STAT1 transcription factor mRNA levels which is one of the well-defined factors in mediating IFN responses^2, 35^. The antiviral roles of chIFN-κ, potentially mediated by the ISGs, were identified in different primary cell/organ models that include *in vitro* primary cell culture and *ex vivo* tracheal culture systems. Intriguingly, profiling of DF1 cells, which are immortalized chicken fibroblasts, failed to show significant induction of these ISGs suggesting cell-specific roles of chIFN-κ. Although, all type I IFNs regulate several similar biological activities, they exhibit significant levels of differences in their potency and immunoregulatory activities^1, 4, 35–37^. These differential effects in signalling along with their spatial and temporal expression determine the functional specificities for each subgroup of type I IFN. Understanding those factors that define the targeted effects of different type I IFNs in a tissue-specific manner would be a fertile area of future research.

It has been shown that mammalian IFN-κ is expressed in unstimulated keratinocytes^11^ and certain lymphoid cell populations such as dendritic cells and monocytes^38^, and the antiviral effects are likely to trigger immune responses against pathogens that infect skin^39^. In contrast, other known type I IFNs express predominantly in virus-infected cells and are not expressed in unstimulated keratinocytes^40, 41^. This suggests that IFN-κ may play crucial and novel roles in skin defence system possibly against cellular challenges and skin-infecting pathogens. Despite skin-specificity, ectopic expression of chIFN-κ and stimulation of non-keratinocytes (chicken fibroblasts) inhibited the replication of RNA viruses (AIV and NDV) which are not specialized skin-infecting pathogens. Further studies would be required to understand whether the antiviral potential of chIFN-κ is stronger in keratinocytes against skin-infecting viruses such as herpesviruses and papillomaviruses, especially when chIFN-κ shown weaker virus-inhibitory effects than chIFN-β in fibroblasts. However, as is reported earlier^11, 42^ for IFN-κ in mammals, there was no detectable expression of chIFN-κ gene in liver, kidney, spleen, beak, trachea, lung and duodenum collected from influenza-infected chickens. In contrast, noticeable expression of chIFN-κ gene was detected in the skin collected from healthy chickens and in herpesvirus (MDV)-infected primary fibroblasts, splenocytes and immune cells (CD4 naïve) (Fig. S1A). However, due to unavailability of reliable chicken keratinocytes cell line in the facility, it requires to determine the antiviral potential and mechanisms of innate immune induction against viruses that infect skin.

Functional investigations and structural similarities of chIFN-κ promoter region indicate that chIFN-κ may share functional similarities to the corresponding IFN-κ proteins from other species. Similar to type I IFNs, transcription factors including IRFs, ISRE and NF-κB binding sites were predicted in the proximal promoter region of the chIFN-κ. Additionally, several GAAANN elements were identified which are fundamental for the virus-induced expression of the IFNs^24, 26^. These sites are characteristics for type I IFNs, however, due to differential positions, it may likely be that chIFN-κ regulates transcriptional activation of innate immune genes different than rest of type I IFNs in a tissue or stimuli-specific manner. While the transcription factors binding sites are identified in this less characterized class of type I IFNs, it is now required to investigate the interaction between regulatory elements and transcription factors in IFN-κ mediated innate immune regulation, and to determine the comparative potency and range of cellular activities by IFN-κ and rest of the type I IFNs in the host defence and cellular maintenance.

Our attempts to transgenically overexpress chIFN-κ yielded strong evidence that this cytokine possess antiviral activities also in developing embryos. We used competent retroviral gene transfer vector system, RCAS^21, 32^ to generate mosaic transgenic chicken embryos that stably and constitutively overexpress chIFN-κ. As part of replication cycle of the retrovirus, the foreign genes are potentially integrated into the host genomes allowing stable expression of the transgenes over the lifetime of the host/cells^21^. This gene-delivery property of RCAS can be exploited to deliver and express genes *in ovo* at the early embryonic developmental stages to generate transgenic embryos in studying desirable phenotypes^21, 32, 33^. While wild type RCAS appeared to be safe, the transcriptional activation of innate immune genes by chIFN-κ as well as chIFN-β established a profound hostile environment for the replication of RNA viruses. Given that embryo mortalities were observed independent of RCAS-infectivity and that transgene-induced antiviral state was incapable to inhibit retroviruses, the RCAS system appeared to be an effective way of delivering genes in developing embryos to study both the impact of transgenes on embryo development and to investigate the transgenes-induced phenotypes. Non-deleterious effects of IFNs on RCAS viruses might be attributed to the continuous expression of proteins from genomic insertions instead of expression during the replication of retroviruses. However, there remain to evaluate the effect of RCAS viruses on the embryo developments, survivability and alterations in cumulative host responses against challenged viruses.

Taken together, the present study provides genetic insights and expands our knowledge into the type I IFN repertoires in avian species and offers functional data on the interaction of the chIFN-κ with RNA viruses of poultry health importance. In an ongoing functional study, we are establishing chicken keratinocyte primary cell line for chIFN-κ induced transcriptional profiling using transcriptomics and proteomics approaches, and to investigate the comparative antiviral roles of chIFN-κ against skin infecting avian herpesviruses (i.e. MDV).

## Material and Methods

### Data mining and bioinformatics analysis

Chicken genome (Ensembl) and expressed sequence tags (EST) databases were screened for the homologues of IFN family gene members using the BLAST algorithm. A stretch of sequence showing high sequence similarity to type I IFN was identified, and characterized to reveal possible open reading frame (ORF). The IFN-κ coding region was amplified from chicken genomic DNA using oligonucleotides primers (GGA TCC GCC ACC ATG TAT GCT TTT GGC TTT ATA CAA ATT GGC TTC A and GAA TTC TTA CTT GTA AAG CTT CCT TAT GAC TTT ATC AAT CAA) that correspond to nucleotide 993–1026 and 1511–1546 in Fig. 1. The genomic nucleotide sequence of IFN-κ including the promoter region was amplified using primers chIFNk-prom-XholR: AAC CCT CGA GGG TAA AGA TAA GCT GCT GTG CTC CTC CTT TCC TAT A and chIFNk-prom-KpnIF: GGA GGG GTA CCC AAT GTA TCT GTA TAT TGT GTA TAG TAT AC. The ORF and homology searches for IFN-κ were carries out in the ORF Finder programme (http://www.ncbi.nlm.nih.gov/projects/gorf) and BLAST tool (http://www.ncbi.nlm.nih.gov/BLAST/) integrated in the NCBI database. Possible transcription start site was identified using promoter predictor programme (http://www.fruitfly.org/seq_tools/promoter.html) whereas potential transcription factor binding sites were predicted using the MatInspector server (http://www.genomatix.de). Signal peptide and protein domains were identified in SMART (http://smart.embl-heidelberg.de/) and SignalP 4.1 Server (http://www.cbs.dtu.dk/services/SignalP/). The IFN sequences from aves and non-aves were acquired from NCBI and aligned using ClustalW programme. The phylogenetic analysis was performed using neighbour-joining method with bootstrap value of *n* = 2,000 in the MEGA programme version 6^43^. Three-dimensional structure of chIFN-κ was determined using I-TASSER Suite^44^ and annotated using MacPyMOL.

### Viruses

Low pathogenicity avian influenza virus (A/chicken/Pak/UDL-01/08/H9N2, abbreviated as UDL/08/H9N2 and Newcastle disease virus (NDV, strain LaSota) were produced in embryonated chicken eggs as described before^45–47^. The UDL/08/H9N2 was titrated by plaque assay in Madin-Darby canine kidney (MDCK) cells and expressed as plaque-forming units (PFU). The NDV-LaSota strain was quantified using immunostaining and expressed as focus-forming units (FFU). Vesicular stomatitis virus (VSV) expressing green florescent protein (VSV-GFP) was kindly provided by Dennis Rubbenstroth (Institute for Virology, Medical Centre – University of Freiburg, Germany). VSV-GFP was propagated and quantified in DF-1 cells and were represented in FFU.

### Cells, media and reagents

Chicken embryo fibroblasts (CEFs) were prepared from 9 day old embryonated eggs at The Pirbright Institute as described previously^48^. CEF, DF-1 (immortalized chicken fibroblasts) and MDCK cells were maintained in Dulbecco’s Modified Eagle Medium (DMEM) supplemented with 10% foetal bovine serum (FBS), 1% penicillin and streptomycin (P/S) at 37 °C in 5% CO_2_ incubator. The Drosophila Schneider 2 (S2) cells were maintained in the Schneider’s Insect Medium (Sigma-Aldrich, Dorset, UK). AMV-3C2-S (gag) antibodies were purchased from Hybridoma Bank of Iowa, University of Iowa. Antibodies against nucleoprotein (NP) of influenza virus and the fusion (F) protein of NDV were raised in mouse as described previously^49, 50^. Alexa-flour 568 secondary antibodies were purchased from Invitrogen Carlsbad, CA, USA and IRDye 800CW α-mouse secondary antibody were acquired from LI-COR, Nebraska USA.

### Expression vectors

The full-length chicken IFN-κ ORF (Met^1^-Lys^184^) was PCR-amplified using primers that were tailed with 5′ BamHI site, 3′ EcoRI site and a consensus Kozak translation sequence (CCACCATG). The BamHI and EcoRI digested amplicons were subcloned in the mammalian expression vector, pcDNA™3.1^(+)^ (Invitrogen, Carlsbad, CA, USA), which contains a human cytomegalovirus immediate-early promoter and bovine growth hormone polyadenylation signal sequence. Correspondingly, the ORF for chIFN-β (Accession Number, NM_001024836) was cloned in pcDNA3.1+ and final constructs were labelled as pcDNA3.1-chIFN-β, and pcDNA3.1-chIFN-κ. Additionally, mature peptide sequences for IFN-κ (Leu^22^-Lys^184^) and chIFN-β (Cys^28^-Gln^203^) were 5′ flanked with the Drosophila BiP signal sequence whereas at the 3′ end, 6xHis and V5 tags were separated from IFN-κ sequence by Tobacco Etch Virus (TEV) protease cleavage site. The entire cassette was chemically synthesised and subcloned between EcoRI and SacII restriction sites in the insect S2 vector system, pS2V1. Final constructs were labelled as chIFN-β_S2 and chIFN-κ_S2. All clones were sequenced from both ends for correct frame and orientation.

### Expression and purification of chIFN-β and chIFN-κ proteins

For expression of chIFN-β and chIFN-κ proteins in heterologous mammalian system, HEK-293 cells were transfected with 25 µg each of pcDNA3.1-chIFN-β, and pcDNA3.1-chIFN-κ or empty pcDNA™3.1^(+)^ vectors using Lipofectamine 2000 (Invitrogen, Carlsbad, CA, USA) and selected with G418 (500 μg/ml) antibiotics. Stable cell clones for chIFN-β and chIFN-κ were expanded and grown in reduced serum (2%) conditions. Supernatants, collected twice each after 24 hours of stable cell growth, were pooled, cleared and concentrated using Centricon 70 columns (Millipore, Billerica, MA, USA). IFN titers were determined using VSV-GFP bioassay (see below) and aliquots were stored at −80 °C until use.

To compare the nature of chIFN-β and chIFN-κ proteins expressed in non-avian and non-mammalian system, Drosophila Expression System (DES®, Invitrogen, Carlsbad, CA, USA) was used. Insect S2 cells (5 × 10^6^ in T25 flask) were transfected with chIFN-β_S2, chIFN-κ_S2 or empty pS2V1 vectors using Calcium Phosphate (Invitrogen, Carlsbad, CA, USA) as per manufacture’s recommendations. Stable cell lines were established using zeocin (1500 µg/ml, Invivogen, San Diego, California, USA) antibiotic selection. Supernatants from serum-deprived stable clones were collected, pooled and cleared from debris using low centrifugation. IFN titers were determined using VSV-GFP bioassay and aliquots were stored at −80 °C until use.

### VSV-GFP IFN bioassay

IFN-induced protection against VSV-GFP was used to identify IFN-producing stable clones and to quantify IFNs preparations, as described before^51^. Briefly, DF-1 cells were seeded in 96-well plates until they are 90% confluent followed by treatment with serial dilutions of supernatants-containing IFNs (from HEK-293 and S2 cells) for 24 hours. These IFN-stimulated cells were infected with VSV-GFP with an MOI of 1. After 24 hours post-infection, VSV-GFP replication was correlated with the change in GFP fluorescence signal intensities using Luminometer (Promega, Madison, WI, USA). The percentage antiviral activities of IFNs were determined by comparing percentage reduction of corrected GFP signal intensities (subtracted background fluoresce signal intensities of uninfected control from GFP signal intensities of IFN treated and virus infected wells) with the mock treated and VSV-GFP-infected control wells. One unit (U) of IFN in the supernatants-containing IFNs was defined as volume of the preparation carrying 50% inhibitory activity against VSV-GFP. Quantity of IFNs (1000 U/ml) collected from serum-deprived HEK-293 stable cell lines was used throughout the study to monitor the antiviral effects of chIFN-κ and chIFN-β. To exclude the possibility of antiviral effects of other secretory cytokines in cell culture, the supernatants from mock-transfected wells was removed as background virus-interference effects.

### Preparation of tracheal organs culture and viral inhibitory effects of IFNs

Tracheal organs culture (TOCs) were prepared from 20 days old chicken from SPF flocks as described before^52^. TOCs with active ciliary movements were used for experimentation. TOCs were primed with indicated amount of IFNs for 24 hours before infection with UDL/08/H9N2 virus. Individual TOCs were collected and fixed on paraformaldehyde blocks for sectioning. Sections were mounted on coverslips, stained with anti-mouse NP, Alexa-flour 568 secondary antibodies and 4′, 6-diamidino-2-phenylindole. Culture media harvested from infected TOCs was subjected to plaque assays for virus quantification^46^.

### Quantitative reverse transcriptase PCR

Total RNA was extracted from IFNs-stimulated DF1 or CEFs using TRIzol reagents (Invitrogen, Carlsbad, CA, USA). A total of 200 ng of RNA was used in PCR reactions using SuperScript® III Platinum® SYBR® Green One-Step qRT-PCR Kit (Invitrogen, Carlsbad, CA, USA). The abundance of specific mRNA was compared to the 28 S rRNA (GGCGAAGCCAGAGGAAACT and GACGACCGATTTGCACGTC) in the Applied Biosystems Prism 7500 system. The reaction was carried out in ABI 7500 light cycler using the following thermo profile; 50 °C for 5 minutes hold, 95 °C for 2 minutes hold, followed by 40 cycles of 95 °C for 3 seconds and 60 °C for 30 seconds. Melting curve was determined at 95 °C for 15 seconds, 60 °C for 1 minute, 95 °C for 15 seconds and 60 °C for 15 seconds. Following primers were used for: *OAS* (5′-AAG AAC TGG GAC TTG GTG GC-3′ and 5′-CCT TCA GCT CCC AGA CTG TG-3′), *MDA5*: 5′-AAG ATG AAG CAG AGG GCA GA-3′ and 5′-CCA CTG CCT GTA GGG AGA CA-3′), *Mx*: (5′-CAC TGC AAC AAG CAA AGA AGG A-3′ and 5′-TGA TCA ACC CCA CAA GGA AAA-3′), *STAT1* (5′-ACT GCA TGC ATT GGT GGC CCA-3′ and 5′-GCT GAC GAA CTT GCT GCA GGC-3′) and *IFIT5* (5′-CAG AAT TTA ATG CCG GTT ATG CAA-3′ and 5′-TGC AAG TAA AGC CAA AAG ATA AGT GT-3′).

### In-cell Western blotting

The DF-1 cells, transfected with the RCASBP(A)–chIFN-β, RCASBP(A)–chIFN-κ or empty RCASBP(A)-wt vectors, were maintained and expanded as described below. A total of 2500 cells were seeded per well in the 96 wells plate for 24 hours, followed by infection with UDL/08/H9N2 (MOI of 1) or were left untreated. After virus adsorption for 1 hour, cells were maintained in the maintenance media for 24 hours. Cells were washed, fixed in 4% paraformaldehyde, and permeabilized with 0.1% TritonX, followed by staining with the influenza virus NP protein-specific monoclonal antibodies and IRDye 800CW α-mouse secondary antibody (LI-COR, Nebraska USA). The 96-wells plate was then imaged using the Odyssey CLx Imaging System (LI-COR, Nebraska, USA).

### Plaque assays

Allantoic fluids and infectious viruses from cell culture and TOCs supernatants were titrated by plaque assays on MDCK cells. Briefly, MDCK cells were inoculated with 10-fold serially diluted samples and overlaid with 0.6% agarose (Oxoid, Hampshire, UK) in overlay DMEM (1× MEM, 0.21% BSA V, 1 mM l-glutamate, 0.15% sodium bicarbonate, 10 mM HEPES, 1× penicillin/streptomycin (Gibco, Carlsbad, CA, USA) and 0.01% Dextran DEAE, with 2 µg ml^−1^ TPCK trypsin (Sigma-Aldrich, Dorset, UK). Plates were incubated at 37  °C for 72 h and were developed using crystal violet stain containing methanol.

### Luciferase assays

To determine responsiveness of Mx promoter to chicken IFNs, chicken fibroblasts grown in 96-well plate format at 2 × 10^4^ to 4 × 10^4^ cells/well, were co-transfected with 150 ng/well of pGL3-P-chMx-luc (Kindly provided by Nicolas Ruggli, Switzerland) and 10 ng/well of a plasmid constitutively expressing Renilla luciferase (phRL-SV40; Promega, Madison, WI, USA) using Lipofectamine 2000^53^. After 24 hours of transfection, cells were stimulated with serial dilution of chIFN-κ, chIFN-β or left unstimulated. Cells were then lysed using 20 μl of 1× passive lysis buffer (Promega, Madison, WI, USA), and samples were assayed for Firefly and Renilla luciferase activity using the Dual-luciferase Reporter Assay System by following supplier instructions and Luminometer (Promega, Madison, WI, USA).

### Construction of chIFN-β, chIFN-κ and eGFP expressing RCASBP(A) vectors and rescue of recombinant RCASBP(A) viruses

The putative coding regions (signal and mature peptide sequences) of the chIFN-β and chIFN-κ were amplified from RNA extracted from the NDV-infected primary CEFs and from chicken genomic DNA, respectively. The amplified products were subcloned into an improved version of RCASBP(A)-ΔF1 (kindly provided by Stephen H. Hughes, National Cancer Institute, MD, USA) *via* the ClaI and MluI restriction sites which replace the *src* gene while maintaining the splice accepter signals. The resultant constructs were names as RCASBP(A)–chIFN-β and RCASBP(A)–chIFN-κ. Similarly, GFP encoding RCASBP(A), referred as RCASBP(A)-eGFP, was generated by introducing the coding sequence of the GFP in between the ClaI and MluI sites. The insert gene orientation and sequence validity was confirmed by DNA sequencing.

To rescue recombinant RCASBP(A) viruses, a total of 2.5 × 10^5^ DF-1 cells were seeded in 25 cm^2^ flasks and maintained at 37 °C, 5% (vol/vol) CO_2_ for 24 hours (~80% confluent). Cells were washed with PBS and were transfected with 2.5 μg of each of the RCASBP(A)-eGFP, RCASBP(A)–chIFN-β and RCASBP(A)–chIFN-κ plasmids using Lipofectamine 2000 in OptiMEM with a pre-determined optimized ratio of 1:6 (Invitrogen, Carlsbad, CA, USA). Media were changed 6 hours post-transfection and the cells were maintained in DMEM supplemented with 10% FCS and 1% penicillin/streptomycin for 48 hours. Expression of the reporter gene (GFP) was monitored using fluorescence microscopy. The GFP-expression confirmed cells were split into 25 cm^2^ flasks and were passaged again into 75 cm^2^ flasks after maintaining for 3 days in-between splits. Finally cells were expanded into 150 cm^2^ flasks until desired number (10^6^ cells/egg) was achieved.

### Generation of mosaic transgenic chicken embryos

#### Experiment 1: Replication dynamics of RCASBP(A) viruses

Eggs (*n* = 20) from specific pathogen free (SPF) Line 0 chicken^54^ were maintained in Biosafety Levels 2 laboratory conditions. A total of one million RCASBP(A)-eGFP infected DF-1 cells (in a volume of 100 μl) were inoculated per egg close to the yolk sac of developing embryos at the age of 3 days post-embryonation using 21 gauge needle through the air sac. The shell fenestration was sealed with sterile autoclave tap. Eggs were kept still for 2 hours before incubation on rotation for the rest of the experiment. During this period, it was expected that the RCAS virus particles are released from the infected DF-1 cells to evade the developing embryo and the viral DNA will integrate into the host genome to complete the retroviral life cycle^21^. A total of three embryos were collected at day 1, 3, 5, 6 and 11 post-RCASBP(A)-eGFP infection. To monitor the expression dynamics of the reporter gene, the whole embryo or selected organs of the embryo were collected at the indicated time points, and were sectioned, DAPI stained and visualize under confocal microscopy for the expression of the GFP.

#### Experiment 2: IFNs-mediated antiviral resistance

Mosaic-transgenic chicken embryos were generated by inoculation of one million RCASBP(A)-chIFN-β, RCASBP(A)–chIFN-κ and wt-RCASBP(A)- infected DF-1 cells at day 3 post-embryonation or were left un-infected as described above. At day 9 post-embryonation (6 days post-infection), each egg was left unchallenged or was challenged with 100 PFU of UDL/08/H9N2 or FFU of NDV-LaSota per egg. Embryo mortality was monitored daily and allantoic fluid was harvested at 14 days of embryonation and was subjected to the plaque assay for virus quantification.

### Statistical analysis

Pairwise comparisons of IFN-treated and control groups were performed using Student’s *t* test. Multiple comparison of treatment groups were analysed using one-way analysis of variance (ANOVA). All statistical tests were conducted in the GraphPad Prism (GraphPad Software, La Jolla, CA, USA).

### Ethics Statement

All animal studies and procedures were carried out in strict accordance with the guidance and regulations of European and United Kingdom Home Office regulations under animal risk assessment numbers AR000695 and AR000733. As part of this process the work has undergone scrutiny and approval by the ethics committee at The Pirbright Institute.

### Nucleotide sequence accession numbers

The chIFN-κ sequences from eight different chicken lines are available under accession numbers from KR817814 to KR817821.

## Electronic supplementary material

Supplementary Material
