# Supplementary material for: Chicken IFN Kappa: A Novel Cytokine with Antiviral Activities
Source: Sci Rep. 2017 Jun 2;7:2719. doi: 10.1038/s41598-017-02951-2 (PMC5457445; doi:10.1038/s41598-017-02951-2)
Supplement: Supplementary file 1 — Supplementary Material [file 41598_2017_2951_MOESM1_ESM.pdf]

Supplementary Material

Chicken IFN Kappa: A Novel Cytokine with Antiviral Activities

Diwakar Santhakumar, Munir Iqbal, Venugopal Nair, Muhammad Munir*

The Pirbright Institute, Woking, Surrey, GU24 0NF, United Kingdom

*muhammad.munir@pirbright.ac.uk
drmunir.muhammad@gmail.com

Supplementary figure legends:

Figure 1S. Expression of chIFN-κ gene in MDV-infected chicken cells and structure of type I IFN locus. (**A**) Transcriptomic profiling of chicken primary fibroblasts and immune cells that were infected with RB1B, Rispens and 675A strains of MDV. Blue bar represents the transcript in the current chicken genome assembly whereas the red bar represents the mappability of the transcript to the chicken genome. Transcripts from MDV-infected data are shown at the bottom. (**B**) Analysis of the type I IFN locus indicates that the chIFN-κ gene clustered outside (~ 27 Mb) the rest of the type I IFN locus on the reverse strand of the chromosome Z. Positions of the IFN-κ and rest of type I IFNs (chIFN-β and chIFN-α) genes represent the coordinates in the Ensambl (Chicken Genome Assembly 5.0).

Figure 2S. Bioinformatics analysis of the chIFN-κ gene. (**A**) Phylogenetic comparison of the chIFN-κ gene with IFN-κ genes from 25 different avian species. The tree was constructed in Mega6 using neighbour-joining methods with boostrap value of 2000. The chIFN-κ gene clustered with corresponding putative IFN-κ genes from other avian species distant from the rest of type I IFNs, and type II and III IFNs of chicken. (**B**) Protein sequence alignment of the chIFN-κ with the IFN-κ from human and bat (*P. alecto*). The chIFN-κ proteins shown amino acid identity throughout the length of the sequence. (**C**) Protein sequence alignment of a representative IFN protein from all known types of IFNs in chicken. Higher protein similarity is depicted among chIFN-κ and chIFN-β and chIFN-α. (**D**) Percentage sequence identity (amino acid) of chIFN-κ with the chIFN-β, chIFN-α, chIFN-λ and chIFN-γ.

Figure 3S. Rescuing and propagation of RCASBP(A) viruses in DF-1 cells and profiling of ISGs in RCASBP(A)-infected chicken cells. (**A**) DF-1 cells monolayer was transfected with the 5ug of RCASBP(A)-eGFP plasmid in one well of 6 wells plate, and rescue and propagation of viruses were monitored for several passages. The expression of the eGFP indicates the progressive replication and infection of cells with the recombinant RCASBP(A)-eGFP viruses. (**B)** Real-time PCR quantification of ISGs (Mx and IFIT5) expression in RCASBP(A) infected cells. The eGFP expressing RCASBP(A) viruses didn't induced a significant level of ISGs compared to chIFN-κ and chIFN-β expressing RCASBP(A) viruses, suggesting its safety to deliver innate immune genes in cells.

A

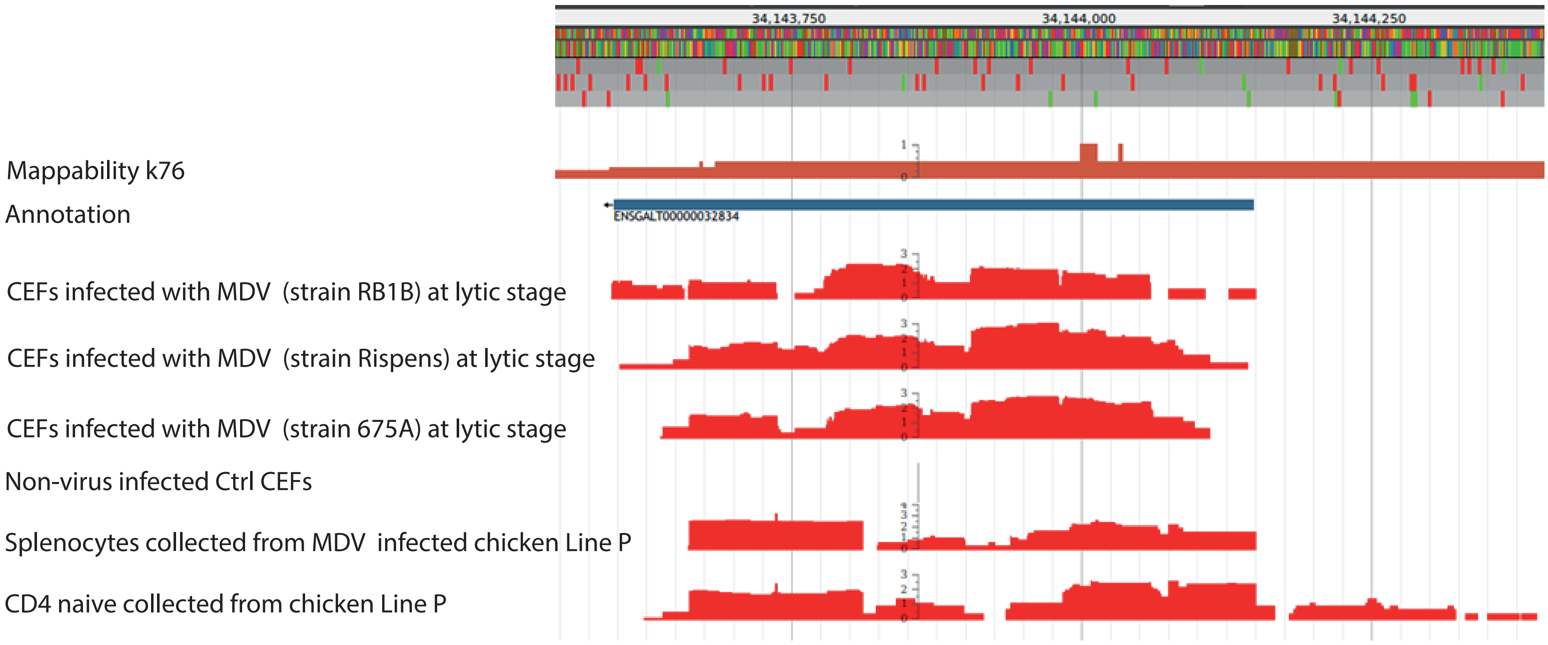
34,143,750
34,144,000
34,144,250
Mappability k76
Annotation
ENSGALT00000032834
CEFs infected with MDV (strain RB1B) at lytic stage
CEFs infected with MDV (strain Rispens) at lytic stage
CEFs infected with MDV (strain 675A) at lytic stage
Non-virus infected Ctrl CEFs
Splenocytes collected from MDV infected chicken Line P
CD4 naive collected from chicken Line P

B

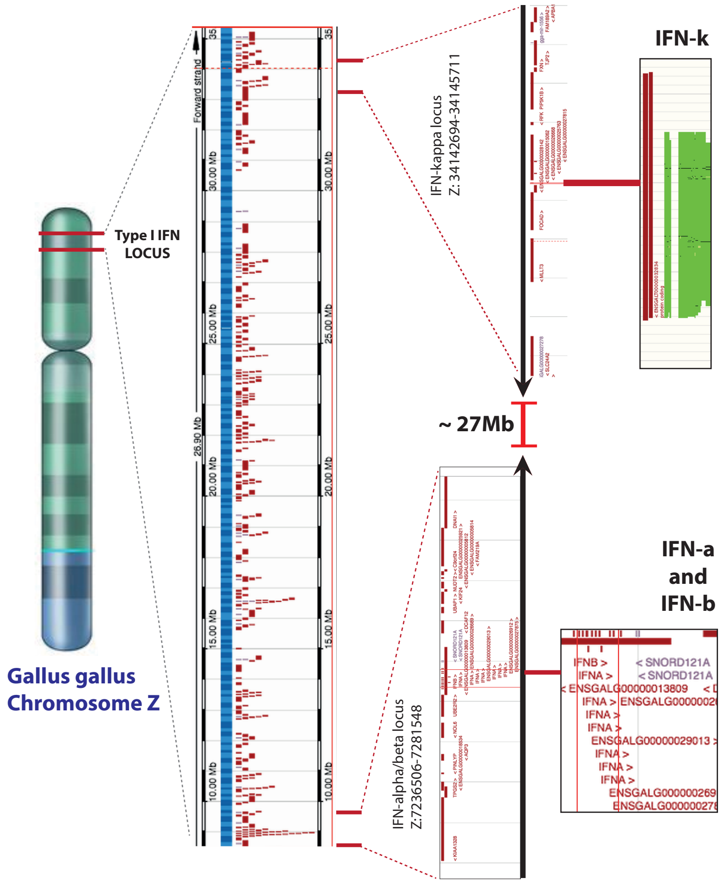
Type I IFN LOCUS
Gallus gallus Chromosome Z
Forward strand
IFN-kappa locus Z: 34142694-34145711
IFN-k
~ 27Mb
IFN-a and IFN-b
IFN-alpha/beta locus Z:7236506-7281548
IFNB >
IFNA >
< SNORD121A
< SNORD121A
ENSGALG00000013809
ENSGALG00000029013 >

A

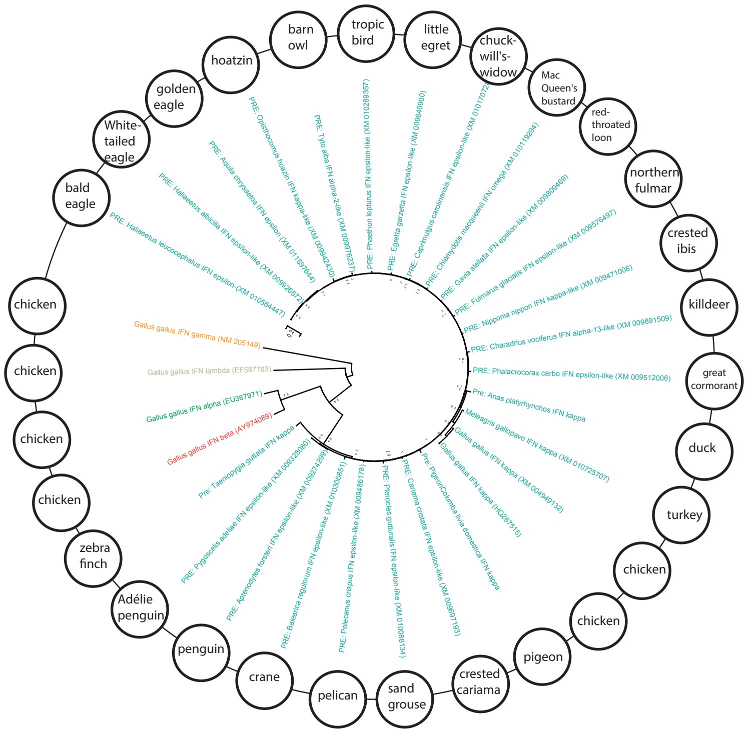

B

```
Gallus gallus IFN_beta     --MTANHQSPGMHSILLL-LLLPALTTTFS-CNHLRHQDANFSWKSLQLLQNTAPPPPQPCPQQDVTFPF 66
Gallus gallus IFN_kappa    --------MYAFGFIQIGFILLCTITISSLTCNHLPLQQRRVIESSLQLLDKMGRRFPQQCLREKMSFRF 62
Gallus gallus IFN_gamma    -------MTCQTYNLFVLSVIMIYYGHTASSLNLVQLQD-----DIDKLKADFNSSHSDVADGGPIIVEK 58
Gallus gallus IFN_alpha    MAVPASPQHPRGYGILLLTLLLKALATTASACNHLRPQDATFSHDSLQLLRDMAPTLPQLCPQHNASCSF 70
Gallus gallus IFN_lambda   ------MVCYGVTIILVGTLGSLLVGAFPQVTPKKSCSLSKYQFPAPLELKAVWRMKEQFEDIMLLTNRK 64

Gallus gallus IFN_beta     PETLLKSKDKKQAAITTLRILQHLFNMLSSPHTPKHWIDRTRHSLLNQIQHYIHHLEQCFVNQGTRSQRR 136
Gallus gallus IFN_kappa    PEQVLKPRQKETVKVAIEEILQHIFYIFSKNLTLAAWDGAALEQFQNGLYQQIEKLEACIIKKQTQYFWS 132
Gallus gallus IFN_gamma    LKNWTERNEKRIILSQIVSMYLEMLENTDKSKPHIKHISEELYTLKNNLPDGVKKVKDIMDLAKLP--MN 126
Gallus gallus IFN_alpha    NDTILDTSNTRQADKTTHDILQHLFKILSSPSTPAHWNDSQRQSLLNRIHRYTQHLEQCLDSSDTRSRTR 140
Gallus gallus IFN_lambda   CNTRLFHRKWDIAELSVPDRITLVEAELD--LTITVLTNPTTQRLAETCQQPLAFLTQVQEDLRDCLALE 132

Gallus gallus IFN_beta     GPRNAHLSINKYFRSIHNFLQHNNYSACTWDHVRLQARDCFRHVDTLIQWMKSRAPLTASSKRLNTQX 204
Gallus gallus IFN_kappa    KEVN-RLKLKKYFQKIDSFLKEKQHNLCSWEISRAEMRRCLQLIDKVIRKLYKX-------------- 185
Gallus gallus IFN_gamma    DLRIQRKAANELFSILQKLVDPP-----SFKRKRSQSQRRCNCX------------------------ 165
Gallus gallus IFN_alpha    WPRNLHLTIKKHFSCLHTFLQDNDYSACAWEHVRLQARAWFLHIHNLTGNTRTX-------------- 194
Gallus gallus IFN_lambda   APSHQPSGKLRHWLQKLETAKKKETAGCLEASAILHIFQVLNDLRCAAQREDCTX------------- 187
```

C

	chIFN-β	chIFN-α	chIFN-λ	chIFN-γ
chIFNκ	25.85	24.87	9.04	8.60
chIFNβ		49.28	10.24	7.80
chIFNα			10.31	10.88
chIFNλ				4.79

A

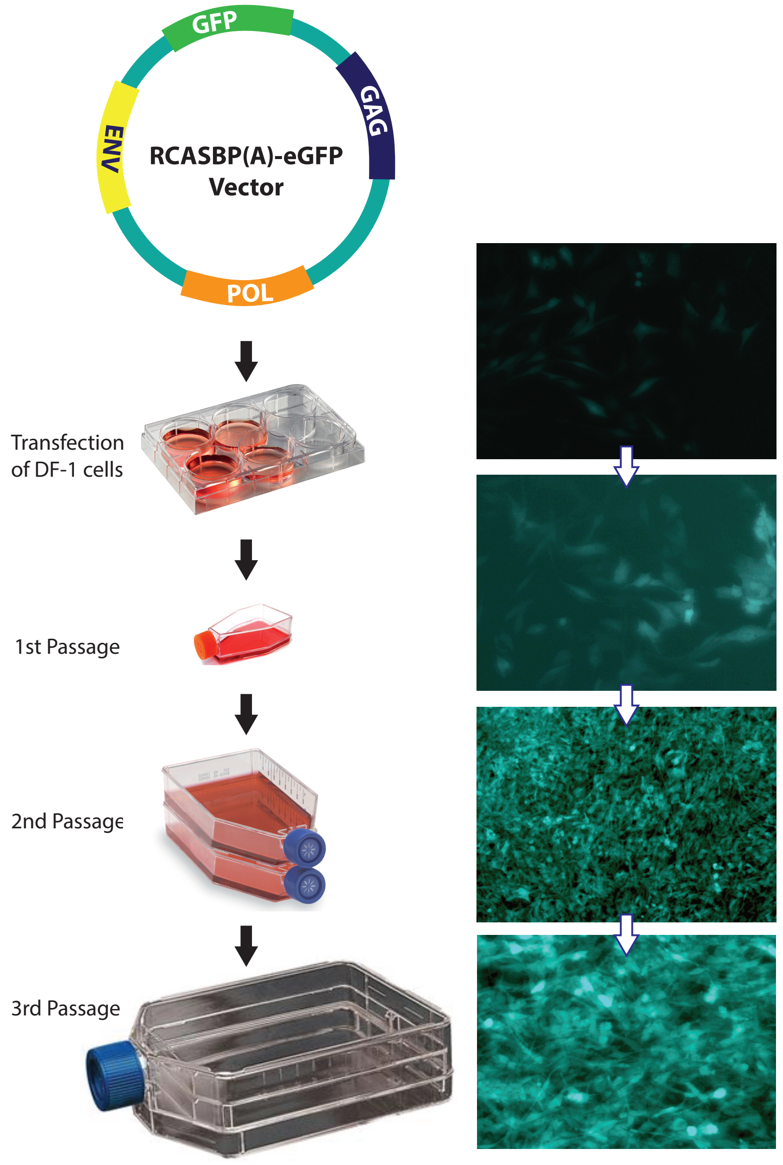

GFP
GAG
ENV
POL
RCASBP(A)-eGFP
Vector
Transfection
of DF-1 cells
1st Passage
2nd Passage
3rd Passage

B

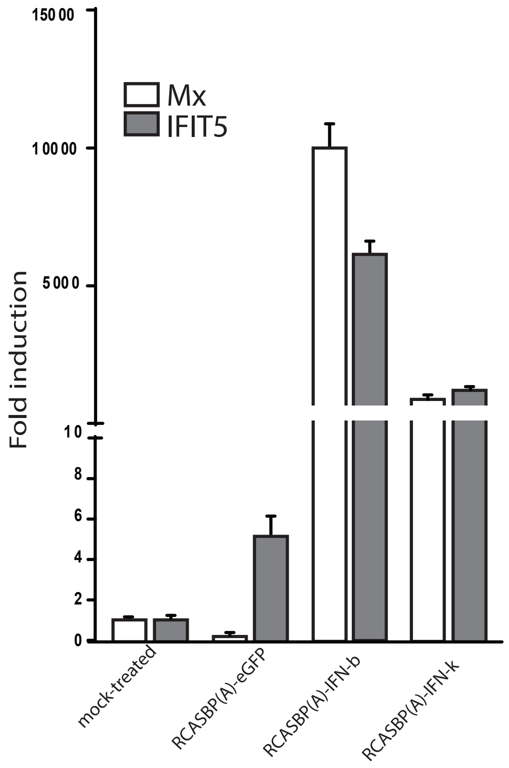

Fold induction
15000
10000
5000
10
8
6
4
2
0
Mx
IFIT5
mock-treated
RCASBP(A)-eGFP
RCASBP(A)-IFN-b
RCASBP(A)-IFN-k
